# Dexamethasone Down-Regulates Expression of Triggering Receptor Expressed on Myeloid Cells-1: Evidence for a TNFα-Related Effect

**DOI:** 10.3389/fpubh.2013.00050

**Published:** 2013-11-14

**Authors:** Ira Mihailidou, Aimilia Pelekanou, Aikaterini Pistiki, Aikaterini Spyridaki, Ira-Maria Tzepi, Georgia Damoraki, Evangelos J. Giamarellos-Bourboulis

**Affiliations:** ^1^4th Department of Internal Medicine, University of Athens Medical School, Athens, Greece; ^2^Integrated Research and Treatment Center, Center for Sepsis Control and Care, Jena University Hospital, Jena, Germany

**Keywords:** TREM-1, TNFα, monocytes, *Pseudomonas aeruginosa*, dexamethasone

## Abstract

**Objectives:** To investigate the effect of dexamethasone on triggering receptor expressed on myeloid cells-1 (TREM-1).

**Methods:** Wild-type and tumor necrosis factor (*TNF*^−/−^) mice were pre-treated with saline, dexamethasone, or hydrocortisone and exposed to a lethal infection of *Pseudomonas aeruginosa*. Mortality and TREM-1 on neutrophil membranes was measured after sacrifice. U937 human monocytic cells were stimulated with lipopolysaccharide (LPS) or heat-killed *P. aeruginosa* without or with dexamethasone or hydrocortisone, and cell-surface TREM-1 and soluble TREM-1 (sTREM-1) were quantified. Expression of TREM-1 and sTREM-1 was also studied in LPS-stimulated U937 cells incubated in the absence or presence of TNFα or anti-TNFα antibody.

**Results:** Pre-treatment with dexamethasone, but not hydrocortisone, prolonged animal survival. Mice pre-treated with dexamethasone showed decreased expression of TREM-1 on neutrophils. In U937 cells, LPS or heat-killed *P. aeruginosa* induced the expression of TREM-1 and the release of sTREM-1. U937 TREM-1 and sTREM-1 were decreased upon addition of dexamethasone but not hydrocortisone. The suppressive effect of dexamethasone was enhanced in the presence of exogenous TNFα and lost in the presence of anti-TNFα antibody. In *TNF*^−/−^ mice, dexamethasone suppression of mortality and TREM-1 neutrophil expression was lost. Gene expression of *TREM-1* in U937 monocytes was decreased after treatment with dexamethasone.

**Conclusion:** TREM-1/sTREM-1 is a novel site of action of dexamethasone. This action is associated with down-regulation of gene expression and is mediated by TNFα.

## Introduction

Triggering receptor expressed on myeloid cells-1 (TREM-1) is a transmembrane receptor that is expressed on the membrane of cells of myeloid origin, notably neutrophils and monocytes. TREM-1 stimulation leads to production of pro-inflammatory cytokines, including tumor necrosis factor-alpha (TNFα) and interleukin (IL)-8. The ligand for TREM-1 has not yet been recognized although it is known that stimulation of myeloid cells with bacterial lipopolysaccharide (LPS) up-regulates TREM-1 expression (1). TREM-1 is also shed in the systemic circulation from cell membranes and the soluble receptor, sTREM-1, behaves as an anti-inflammatory mediator (2).

Current evidence suggests that TREM-1/sTREM-1 may be an important player in the pathogenesis of sepsis. TREM-1 activity is thought to be pro-inflammatory and expression of TREM-1 is elevated on circulating neutrophils in patients with septic shock (3). sTREM-1 is considered to possess anti-inflammatory activity, since injection of sTREM-1 protects mice from lethal endotoxic shock (2). Evidence also suggests an anti-inflammatory role of sTREM-1 in human sepsis, since it follows the kinetics of anti-inflammatory cytokines (4). Therefore, TREM-1/sTREM-1 may be targets for host response modulation in order to offer a survival benefit for the host.

Corticosteroids are the most widely used suppressors of the immune response. Corticosteroids with different glucocorticoid activities have been studied in clinical trials of sepsis with uneven results. Administration of hydrocortisone in sepsis can decrease circulating levels of pro-inflammatory cytokines (5, 6). Dexamethasone is the corticosteroid with the greatest anti-inflammatory activity, and inhibits the *ex vivo* production of TNFα, IL-6, IL-8, and IL-12p40 by LPS-stimulated whole blood from patients with sepsis in a dose-dependent manner (7). However, the effect of corticosteroids on TREM-1/sTREM-1 has never been described.

The purpose of this study was to investigate the effect of corticosteroids on TREM-1 expression on neutrophils in a lethal mouse infection model, and to examine the stimulated expression of TREM-1 and on sTREM-1 on human myeloid cells *in vitro*.

## Materials and Methods

### Animal studies

Eighty-six male wild-type C57BL/6 and 32 male homozygous *TNF* knockout (*TNF*^−/−^) mice weighing 27–29 g were used in the study. The *TNF*^−/−^ mice (Alexander Fleming Institute, Vari, Athens, Greece) were generated on a C57BL/6 genetic background as described (8). The study received permission from the Veterinary Directorate of the Prefecture of Athens according to the Greek legislation in conformance to the 160/1991 Council Directive of the EU. Animals were housed in metal cages and maintained under specific pathogen-free conditions. Room temperature ranged between 18 and 22˚C, relative humidity between 55 and 65%, and the light/dark cycle was 6:00 a.m./6:00 p.m.

*Pseudomonas aeruginosa 2* was isolated from the blood of a patient with acute pyelonephritis and severe sepsis. The isolate was multidrug-resistant (MDR) as defined by Clinical and Laboratory Standards Institute (CLSI) criteria. The isolate was stored in multiple aliquots in skim milk (Oxoid Ltd, London, UK) at −70˚C. Before each experiment, one aliquot was thawed and cultured on MacConkey agar plates (Becton Dickinson, Cockeysville, MD, USA). Single colonies were suspended in Mueller–Hinton broth (Oxoid) and incubated for 12 h at 37˚C in a shaking water bath. The resulting inoculum was washed three times with 0.9% NaCl to remove free endotoxin; it was diluted to 1 × 10^7^ cfu/ml and a final volume of 0.15 ml was administered using intraperitoneal (i.p.) injection under light ether anesthesia.

Animals were randomly assigned into four groups. Two animals per group were studied per day of experiment.

- Control group (*n* = 16); wild-type mice injected i.p. with 0.15 of sterile water 1 h before bacterial challenge.
- Dexamethasone group (*n* = 16); wild-type mice injected i.p. with 1 mg/kg of dexamethasone (Sigma Co, St. Louis, MO, USA) dissolved in 0.15 ml sterile water 1 h before bacterial challenge. The dose of dexamethasone was based on former studies (9).
- Hydrocortisone group 3.6 mg/kg (*n* = 16); wild-type mice injected i.p. with 3.6 mg/kg of hydrocortisone (Sigma Co) dissolved in 0.15 ml sterile water 1 h before bacterial challenge. The dose of hydrocortisone was based on former studies (9).
- Hydrocortisone group 36 mg/kg (*n* = 16); wild-type mice injected i.p. with 36 mg/kg of hydrocortisone dissolved in 0.15 ml sterile water 1 h before bacterial challenge. The dose of hydrocortisone was 10 times greater than the dose administered to mice in the 3.6 mg/kg hydrocortisone group to investigate the dose-effect of hydrocortisone.

Six animals in each group were sacrificed 6 h after bacterial challenge. This time point was selected based on preliminary experiments with 20 mice sacrificed at serial time intervals post bacterial challenge showing that the peak expression of TREM-1 on neutrophils was observed 6 h after bacterial challenge (data not shown). Survival was recorded for the remaining 10 animals of each group at 6-h intervals for 7 days. Mice were sacrificed by intramuscular (i.m.) injection of 25 mg/kg ketamine. An abdominal midline incision was then performed under sterile conditions, and the intestines were displaced to the left to allow blood sampling by puncture of the lower vena cava with a sterile 26G needle. Blood was collected into EDTA-coated tubes (Vacutainer, Becton Dickinson) and centrifuged, and the serum was transferred to a new tube and stored at −70˚C until assayed. TNFα (R&D Inc, Minneapolis, MD, USA) was measured in plasma by an enzyme immunoabsorbent assay (lower limit of detection 15.6 pg/ml). The sedimented pellets that remained after removal of the serum were re-suspended in Phosphate Buffered Saline (PBS, pH 7.2, Biochrom, Berlin, Germany) and the erythrocytes were lysed after adding 1 mM NH_4_Cl. Leukocytes were washed three times with PBS and incubated with mouse anti-TREM-1 antibody PE (R&D Inc) as described below. Leukocyte subsets were analyzed by the EPICS XL/MSL flow cytometer (Beckman Coulter, Miami, FL, USA) after selection for neutrophils by characteristic Forward/Side scattering. Neutrophils from two uninfected (sham) mice were used as negative controls. Expression of TREM-1 was given as % and mean fluorescence intensity (MFI).

In parallel, specimens of liver were aseptically excised and transferred to separate sterile containers. The livers were weighed and homogenized in 1 ml of Mueller–Hinton broth and diluted four consecutive times using serial 1:10 dilutions in 0.9% NaCl. A 0.1 ml aliquot of each dilution was plated onto MacConkey agar plates. Plates were incubated for 48 h at 35˚C, and the number of colonies were counted and multiplied by the corresponding dilution factor. The results were expressed as log10 cfu/g. The minimum detection limit was 10 cfu/g.

### U937 monocyte experiments

One million cells of the U937 human monocytic cell line were inoculated into each well of a 12-well plate (final volume 2.4 ml). The cell culture medium consisted of RPMI 1640 (Biochrom) supplemented with 2 mM of glutamine (Biochrom), 10% Fetal Bovine Serum (Biochrom), 100 U/ml of penicillin G and 0.1 mg/ml of streptomycin (Sigma Co). The cultures were incubated at 37˚C under 5% CO_2_ for 24 h. Cultures were incubated for 1 h with medium alone (control), 10 μM dexamethasone, or 10 μM of hydrocortisone. Following pre-incubation, cells were stimulated by adding 10 ng/ml of *Escherichia coli* O55:B5 (LPS, Sigma) or 1 × 10^6^ cfu/ml of heat-killed *P. aeruginosa 2*. Heat killing was achieved after incubation for 6 h at 65˚C in a shaking water bath, and lack of growth was determined following inoculation in growth media. In separate experiments, U937 cells were pre-incubated with dexamethasone or hydrocortisone as described above, in the presence of either TNFα (100 pg/ml, Sigma) or anti-TNFα antibody (100 pg/ml, Serotec, Marseille, France). Cells were then stimulated with 10 ng/ml LPS. After 24 h of incubation, the cultured cells were transferred to a sterile tube, centrifuged, and the supernatants were transferred to separate tubes and stored until analysis. sTREM-1 levels were measured in the supernatants by ELISA (R&D Inc). The cell pellets were diluted with 1 ml of PBS and 100 μl of the cell suspension was transferred to a new tube. Ten microliters of human anti-TREM-1 monoclonal antibody labeled with phycoerythrin (PE emission 570 mm, R&D Inc) was added to the mixture and incubated for 40 min at 4˚C. The cells were then washed with PBS pH 7.2 and centrifuged, and the cells were analyzed by the EPICS XL/MSL flow cytometer. Unstained cells and antibody isotype controls [cells stained with IgG-PE (Beckman Coulter)] were used as negative controls. Expression of TREM-1 was expressed as the percent of TREM-1-positive cells compared to the entire population of leukocytes gated, and MFI was calculated based on the TREM-1-positive population after subtracting the background measured in the antibody isotype control cells.

### TREM-1 expression analysis

To measure the effect of dexamethasone on expression of the *TREM-1* gene, U937 monocytes were stimulated with 1 × 10^6^ cfu/ml of heat-killed *P. aeruginosa 2* without or with 10 μM dexamethasone for 8 h. After stimulation, the cultured cells were collected and centrifuged, and the supernatant discarded. The cell pellets were treated with consecutive treatments of Trizol (AppliChem, GmbH, Germany) and chloroform, and contaminating DNA was removed by treatment for 30 min at 37˚C with 0.04 U/μl of DNAase (Ambion). To produce cDNA, 1.5 μg of RNA was incubated with 0.4 mM of dNTPs (New England BioLabs, Ipswitch, MA, USA), 1.0 U of RNA-sin (New England BioLabs), 10 mM DTT (New England BioLabs), and 5× reverse transcriptase buffer. In a Mastercycler 5330 (Eppendorf), the RNA was initially incubated for 10 min at 65˚C. One microunit of reverse transcriptase (New England BioLabs) was added and the reactions consecutively incubated at: 10 min at 25˚C; 50 min at 42˚C; and 15 min at 70˚C. Samples without reverse transcriptase were used as blanks, and cDNA was kept at −80˚C until assayed.

Quantitative PCR was performed in 96-well plates with a iQ5 Real Time PCR Detection System (BioRad Laboratories Inc, USA). Quantification of TREM-1 mRNA levels was calculated relative to the housekeeping gene encoding β2-microglobulin. Reactions were performed in a final volume of 20 μl containing 10 μl FluocycleTM II SYBR Master Mix (EuroClone S.p.a-Italy), 7.0 μl PCR-grade water (AppliChem), 1.0 μl cDNA, and 1.0 μl each of the 20 μM forward and reverse gene-specific primers. The reactions were incubated at 95˚C for 5 min, followed by 40 PCR cycles. Each cycle consisted of three steps: denaturation (95˚C for 10 s), annealing (60˚C for 30 s), and extension (75˚C for 30 s). Blanks were subjected to this protocol as well. Melting curve analyses was performed for all genes and confirmed the specificity of the PCR products by the presence of a single peak. PCR products were visualized on 3% agarose gels and stained with ethidium bromide (AppliChem, GmbH, Germany). PCR primers were: TREM-1, sense 5′-TGG TCT TCT CTG TCC TGT TTG-3′; antisense 5′-ACT CCC TGC CTT TTA CCT C-3; β2-microglobulin, sense 5′-ATG AGT ATG CCT GCC GTG TG-3′; antisense, 5′-CCA AAT GCG GCA TCT TCA AAC-3′ (10). The number of transcripts was measured using the PFAFFL equation (11).

### Statistical analysis

Results are presented as means ± SE. Comparisons were done by the Mann–Whitney *U* test with Bonferroni corrections for multiple comparisons. Survival was calculated by Kaplan–Meier analysis; comparisons between groups were performed by the log-rank test. Percent changes after treatment with dexamethasone were calculated by the formula 100 × [Effect of stimulus − Effect of stimulus after dexamethasone treatment]/Effect of stimulus, where stimulus was either LPS or heat-killed *P. aeruginosa*. Any *p* value below 0.05 was considered statistically significant.

## Results

### Dexamethasone effects on mortality and TREM-1 expression in mice

**Figure 1:**
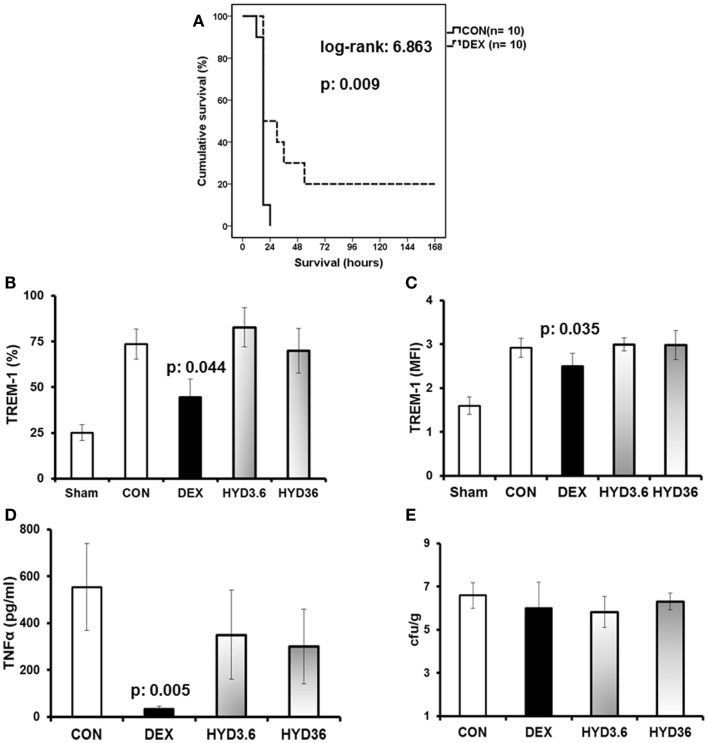
**Dexamethasone is a modulator of TREM-1 expression on mouse neutrophils**. Wild-type male C57BL6 mice were challenged by a multidrug-resistant isolate of *P. aeruginosa*. Ten mice were pre-treated 1 h before challenge either with saline (control group, CON), dexamethasone (DEX), 3.6 mg/kg hydrocortisone (HYD3.6), or 36 mg/kg of hydrocortisone (HYD36). **(A)** Represents survival of the control group (solid line), and dexamethasone-treated animals (dashed line). **(B,C)** Shows the expression of TREM-1 on circulating neutrophils at animal sacrifice, **(D)** shows serum concentrations of TNFα at animal sacrifice, and **(E)** shows liver bacterial loads. Results from **(B–E)** refer to six mice per group. Data in uninfected mice are also provided [Sham in **(B,C)**]. Data for TREM-1 are shown as percent of neutrophils expressing TREM-1 **(B)** or expressed as TREM-1 MFI for neutrophils **(C)**. *P* values indicate significant differences compared to control.

Pre-treatment of C57BL/6 mice with dexamethasone significantly prolonged animal survival (Figure 1A) compared to *P. aeruginosa*-infected mice treated with water (controls). However, pre-treatment with hydrocortisone did not affect survival (data not shown). Compared to control mice that were infected with *P. aeruginosa* but not treated with corticosteroids, the percent of circulating neutrophils expressing TREM-1 was decreased in mice pre-treated with dexamethasone [30% mean reduction, Figure 1B (*p* = 0.044)], and the MFI of TREM-1 on TREM-1-positive cells was also decreased (mean 14.2% reduction, *p* = 0.035). However, pre-treatment of mice with any concentration of hydrocortisone did not significantly affect TREM-1 expression (Figures 1B,C) compared to controls. Administration of dexamethasone decreased serum TNFα (94% mean reduction, *p* = 0.005) but did not affect liver bacterial load (Figures 1D,E).

### Dexamethasone effect on stimulated U937 cells

**Figure 2:**
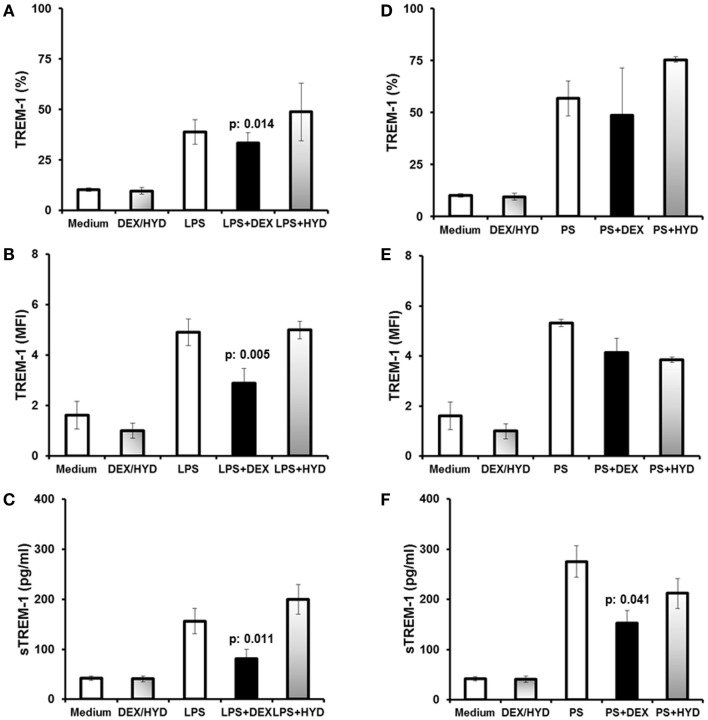
**Dexamethasone is a modulator of TREM-1 expression in human U937 monocytic cells**. Human U937 monocytic cells were stimulated either with *E. coli* lipopolysaccharide (LPS) or with heat-killed *Pseudomonas aeruginosa 2* (PS). U937 cells were treated for 1 h with dexamethasone (DEX) or hydrocortisone (HYD) prior to stimulation. **(A,B)** Show TREM-1 expression after stimulation with LPS, and **(C)** shows sTREM-1 production after stimulation with LPS. **(D,E)** Show TREM-1 expression after stimulation with PS, and **(F)** shows sTREM-1 production after stimulation with PS. DEX/HYD represent unstimulated cells treated only with DEX or HYD, and the results are averaged. Data for TREM-1 are shown as percent of U937 cells expressing TREM-1 **(A,D)**, or as TREM-1 MFI for U937 cells **(B,E)**. Results from six experiments are shown. *P* values indicate significant differences compared to LPS or PS-treated cells.

The human monocytic U937 cell line was utilized to determine the effects of dexamethasone on TREM-1 expression. Cells were pre-incubated without (control) or with dexamethasone or hydrocortisone prior to stimulation with LPS or heat-killed *P. aeruginosa*. Compared to control cells, pre-treatment of U937 cells with dexamethasone prior to LPS stimulation decreased the percent of TREM-1-positive cells by 5.2% (*p* = 0.014, Figure 2A), and dexamethasone pre-treatment reduced the percent of TREM-1-positive cells stimulated with in heat-killed *P. aeruginosa* by 8.2% (not significant, Figure 2D). A similar reduction in TREM-1 MFI was observed in LPS-stimulated cells compared to control cells (Figure 2B, 41.3% mean reduction, *p* = 0.005). There were no significant changes in hydrocortisone-treated cells compared to controls (Figure 2E).

sTREM-1 levels decreased by 51.7% in dexamethasone-treated cells that were stimulated with LPS compared to control cells (Figure 2C, *p* = 0.011). Similarly, sTREM-1 levels decreased by 55.5% in dexamethasone-treated cells that were stimulated with *P. aeruginosa* (Figure 2F, *p* = 0.041). No effect of hydrocortisone was observed on sTREM-1 levels compared to control cells.

### Role of TNFα on dexamethasone suppression of TREM-1 and sTREM-1

**Figure 3:**
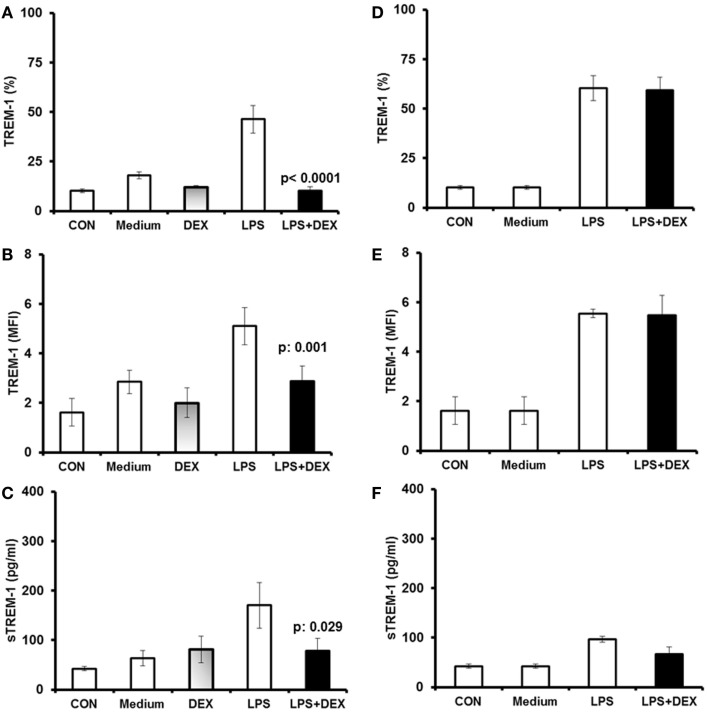
**Dexamethasone modulates TREM-1 expression in human U937 monocytic cells in the presence of TNFα or anti-TNFα antibody**. Human U937 monocytic cells were stimulated with LPS, and expression of TREM-1 on cell membranes and concentrations of sTREM-1 in supernatants were measured after 24 h of incubation. Cells were pre-incubated prior to stimulation for 1 h without or with dexamethasone (DEX) in the presence of either TNFα or anti-TNF blocking antibody. **(A,B)** Show TREM-1 expression after stimulation with LPS in the presence of TNFα, and **(C)** shows sTREM-1 production after stimulation with LPS in the presence of TNFα. **(D,E)** Show TREM-1 expression after stimulation with LPS in the presence of anti-TNFα, and **(F)** shows modulation of sTREM-1 production after stimulation with LPS in the presence of anti-TNFα. CON in **(A–C)** represents unstimulated cells treated in medium alone; CON in **(D–F)** represent unstimulated cells treated in medium alone. Results from six experiments are shown. Data for TREM-1 are shown as percent of U937 cells expressing TREM-1 **(A,D)**, or as TREM-1 MFI for U937 cells **(B,E)**. *P* values indicate significant differences compared to LPS-treated cells.

Since dexamethasone inhibits release of TNFα from whole blood of patients with sepsis (7), we investigated the role of TNFα on the observed effect of dexamethasone inhibition of TREM-1/sTREM-1 expression. Addition of TNFα to U937 cells increased the percent of TREM-1-positive cells stimulated with LPS compared to LPS alone (compare LPS bars in Figures 2A and 3A). Addition of dexamethasone completely suppressed LPS-stimulated production of TREM-1, similar to levels observed for untreated cell controls (Figure 3A, *p* < 0.0001). Similar dexamethasone suppressive effects were observed for TREM-1 MFI (Figure 3B, *p* = 0.001), and sTREM-1 levels (Figure 3C, *p* = 0.029) compared to the LPS-only controls.

To separately confirm the affects of TNFα on TREM-1 expression, U937 cells were incubated with an anti-TNFα antibody. Blockade of TNFα activity by the anti-TNFα antibody completely abolished the dexamethasone-dependent suppression of TREM-1 and sTREM-1 (Figures 3D–F).

### Affect of dexamethasone on *P. aeruginosa*-induced mortality and TREM-1 expression in TNF^−/−^ mice

**Figure 4:**
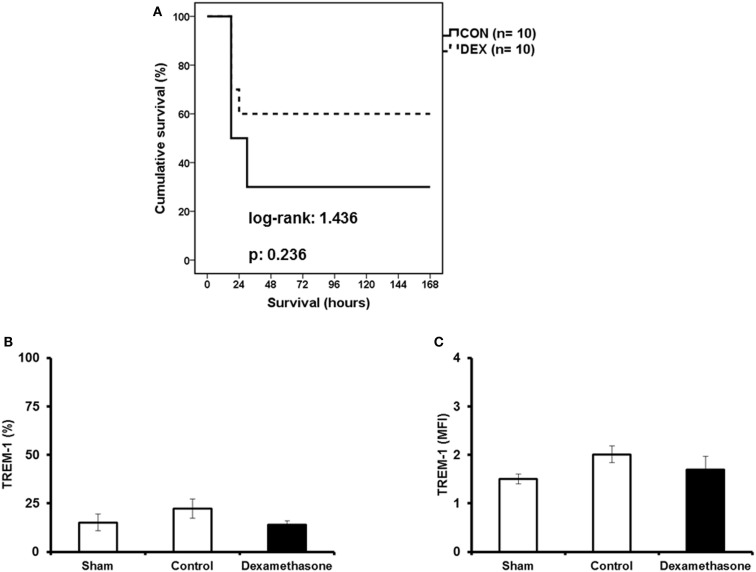
**Dexamethasone is not a modulator of TREM-1 expression in TNF^−/−^ mice**. TNF^−/−^ male mice were challenged by a multidrug-resistant isolate of *P. aeruginosa*. Mice were pre-treated 1 h before challenge with either saline (CON) or dexamethasone (DEX). In **(A)**, survival of 10 control mice (solid line) and 10 DEX mice (dashed line) is shown. In **(B,C)**, expression of TREM-1 on neutrophils at sacrifice performed 6 h after bacterial challenge (six mice per group) is shown. Results from sham-treated mice (Sham, no infection with *P. aeruginosa*) are also provided. Data for TREM-1 are shown as percent of neutrophils expressing TREM-1 **(B)** or expressed as TREM-1 MFI for neutrophils **(C)**.

In order to further understand the significance of TNFα for the suppressive effect of dexamethasone on TREM-1 expression, animal experiments were conducted in mice deficient for *TNF*α. *TNF*^−/−^ mice were pre-treated with either saline (control) or dexamethasone prior to infection with *P. aeruginosa*. As shown in Figure 4A, pre-treatment with dexamethasone did not offer any significant survival benefit. Expression of TREM-1 on circulating neutrophils was not significantly decreased in the dexamethasone-treated mice compared to the control mice (Figures 4B,C).

### Effect of dexamethasone on stimulated TREM-1 gene expression in U937 cells

**Figure 5:**
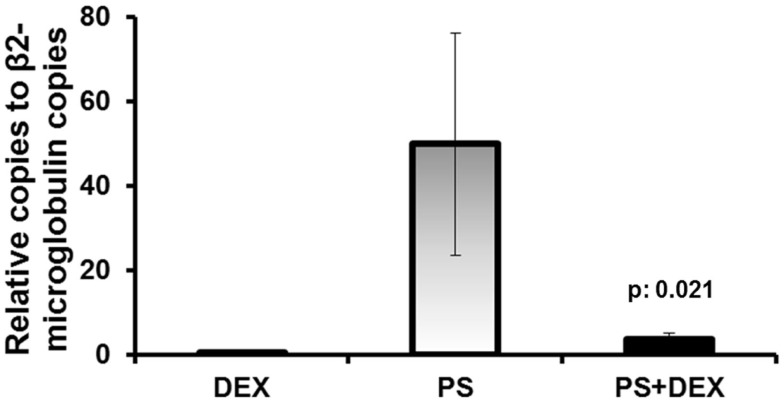
**Dexamethasone down-regulates gene expression of *TREM-1***. Human U937 monocytic cells were stimulated with heat-killed *Pseudomonas aeruginosa* (PS). Cells were pre-treated for 1 h in the absence or presence of dexamethasone (DEX) prior to stimulation. Results from four experiments are shown. *P* value indicates significant difference compared with PS-treated cells.

To examine if the dexamethasone mechanism of action is mediated through an affect on gene expression, U937 monocytes were stimulated with heat-killed *P. aeruginosa*. Treatment of cells with dexamethasone significantly decreased the number of *TREM-1* copies (Figure 5) compared to stimulated cells (92.5% mean reduction, *p* = 0.021).

## Discussion

Our results suggest a new target of action of dexamethasone. Dexamethasone, but not hydrocortisone, down-regulates expression of the inflammation-associated cell-surface receptor TREM-1 on mouse neutrophils and human U937 monocytic cells following stimulation. Dexamethasone also reduces production of stimulated production of sTREM-1. We show that dexamethasone inhibition of stimulated TREM-1 is associated with reduced *TREM-1* gene expression. Finally, we demonstrate that dexamethasone suppression of TREM-1 is dependent on TNFα.

Since TREM-1 expression on cell surfaces has been associated with septic shock in humans (1) and sTREM-1 protects mice from endotoxic shock (2), TREM-1 modulation may have potential as a sepsis therapy. To this end, the presented findings suggest that glucocorticoids may modulate TREM-1. Glucocorticoids may act in septic shock to reverse relative adrenal insufficiency (12, 13). However, corticosteroids are potent anti-inflammatory drugs, and in acute disease settings, may down-regulate the production of monocytic pro-inflammatory cytokines (7). A relationship between corticosteroid administration and sepsis was suggested when the administration of low-doses of hydrocortisone to septic patients significantly reduced circulating levels of IL-6 and of IL-8. IL-6 and IL-8 levels were restored to the former baseline values after the withdrawal of hydrocortisone (5, 6).

Recent evidence generated in a double-blind randomized trial in patients with community-acquired pneumonia suggested a possible clinical role for a dexamethasone-mediated anti-inflammatory effect. Patients were treated intravenously with either placebo or with 5 mg dexamethasone for 4 days. Results from this study indicated a significant reduction of the median length of hospital stay from 7.5 days in the placebo arm to 6.5 days in the dexamethasone arm (14). Two days after the start of treatment, circulating levels of TNFα, IL-6, IL-8, and monocyte chemotactic protein (MCP-1) were decreased compared with placebo-treated patients. The effect of dexamethasone on circulating TNFα levels was more prominent within patients infected by *Streptococcus pneumoniae* (15).

The findings of the present study suggest another site of action of dexamethasone that has not previously been described: TREM-1/sTREM-1. In animal experiments, *P. aeruginosa* was selected for animal challenge since this species is often MDR and a frequent cause for hospital-acquired sepsis. Available antimicrobials are limited for MDR *P. aeruginosa* and management of *P. aeruginosa* infections can be challenging. As a consequence, adjuvant therapies targeting pathogenesis are welcomed. Experimental infection by MDR *P. aeruginosa* led to early death, and pre-treatment with dexamethasone prolonged survival. The dexamethasone affect on survival was accompanied by down-regulation of the expression of TREM-1 on neutrophils. A similar effect was not found for hydrocortisone even when the dose used for pre-treatment was increased 10 times. The greater glucocorticoid biological activity of dexamethasone compared to hydrocortisone may be part of the explanation. The effect of dexamethasone could possibly be extrapolated to infections by other Gram-negative bacteria since down-regulation of TREM-1/sTREM-1 was also shown when LPS was used to stimulate U937 cells. Our studies suggest that dexamethasone-mediated protection observed in the mouse experiments is mediated through down-regulation of *TREM-1* gene expression. Reduced *TREM-1* gene expression also likely explains reduced levels of sTREM-1. The presence of a protease that cleaves cell membrane TREM-1 and releases sTREM-1 has been described on myeloid cells (2). However, we believe it is unlikely that dexamethasone-mediated reduction of sTREM-1 levels results from protease inhibition since cell-surface TREM-1 levels were similarly reduced.

**Figure 6:**
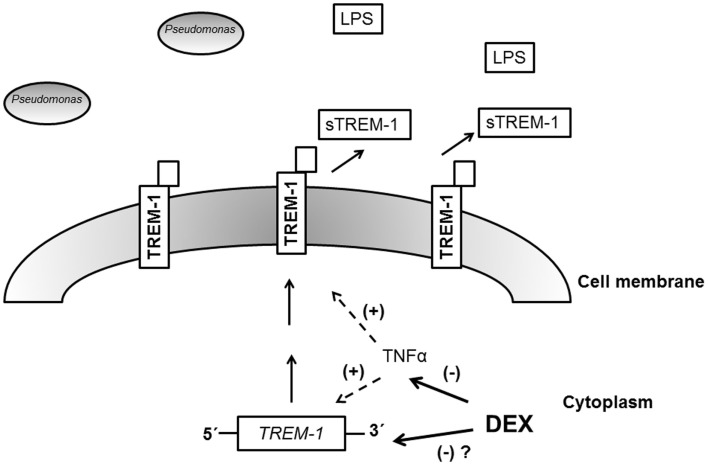
**Model for the mechanism of action of dexamethasone**. After infection with Gram-negative bacteria like *P. aeruginosa*, endotoxin (LPS) induces production of the TREM-1 receptor. Membrane-bound TREM-1 can be proteolytically processed, resulting in release of sTREM-1 into the extracellular space. Dexamethasone (DEX) suppresses the activity of TNFα, and since TNFα may have a positive effect on *TREM-1* expression, DEX indirectly inhibits TREM-1 expression. We speculate that DEX may also directly down-regulate *TREM-1* gene expression, resulting in reduced production of membrane-bound TREM-1 and sTREM-1.

We observed that dexamethasone requires the presence of TNFα to manifest the effect on TREM-1/sTREM-1. *P. aeruginosa* stimulated significant production of TNFα in the mouse model. Further, in studies where TNFα was reduced (either through antibody blockade or through the use of *TNF*^−/−^ mice), the suppressive effect of dexamethasone on TREM-1 expression was lost. The exact relationship between TREM-1/sTREM-1, dexamethasone, and TNFα cannot be fully elucidated with the current data. Since the *TNF* and *TREM-1* genes are located on different chromosomes, down-regulation of *TREM-1* by dexamethasone may require parallel down-regulation of *TNF* (16). A hypothetical mechanism is shown in Figure 6. Alternatively, dexamethasone effect on *TREM-1* gene expression may be dependent on TNF signaling effects.

Two main limitations of this study should be addressed. First, we did not perform dose response experiments with dexamethasone. We do not believe that these experiments were necessary since the applied dose of dexamethasone for both *in vitro* and *in vivo* studies correspond to reported serum levels (9). Second, we did not examine TREM-1 protein levels using Western blot analysis. However, we feel that reporting TREM-1 expression on cells both as percentage and MFI, and measuring of sTREM-1 levels in supernatants adequately supports our conclusions.

Recent findings using mouse sepsis models underscore the possible importance of inhibition of the TREM-1 ligand (as yet undefined) as a strategy for the management of sepsis. In these studies, sepsis was induced after challenge with *Bacillus pyocyaneus* and *P. aeruginosa* (17, 18). Administration of a TREM-1 fusion protein either as pre-treatment 1 h before bacterial challenge or 1 h after bacterial challenge prolonged animal survival. Circulating levels of TNFα, IL-1β, IL-6, and MCP-1 were decreased, whereas tissue bacterial load remained unchanged. Taken together, the findings of these animal studies (17, 18) and results of the present study suggest that modulation of TREM-1 is a possible therapeutic target for acute infection settings. Possible interventions include administration of dexamethasone or a TREM-1 fusion protein. Furthermore, in a recent study by our group, gene transcripts of *TREM-1* and of *TNF* were measured in circulating monocytes from 13 patients with severe sepsis and from seven patients with uncomplicated sepsis within the first 24 h diagnosis. Gene expression of *TNF* in severe sepsis was down-regulated as expected due to the immunoparalysis of circulating monocytes. However, gene expression of *TREM-1* was up-regulated in circulating monocytes of patients experiencing severe sepsis (10). These clinical observations along with the described effect of dexamethasone on down-regulation of *TREM-1* gene expression suggest the possibility of administration of dexamethasone in the septic host.

The presented results indicate TREM-1/sTREM-1 as a novel site of action for dexamethasone modulation. Dexamethasone decreases expression of TREM-1 on myeloid cells, and this phenomenon is related to down-regulation of gene expression and is modulated by TNFα. This action of dexamethasone was accompanied by significant prolongation of survival in experimental infection by *P. aeruginosa*.

## Conflict of Interest Statement

The authors declare that the research was conducted in the absence of any commercial or financial relationships that could be construed as a potential conflict of interest.
